# Constitutive Overexpression of a Conifer *WOX2* Homolog Affects Somatic Embryo Development in *Pinus pinaster* and Promotes Somatic Embryogenesis and Organogenesis in *Arabidopsis* Seedlings

**DOI:** 10.3389/fpls.2022.838421

**Published:** 2022-03-10

**Authors:** Seyedeh Batool Hassani, Jean-François Trontin, Juliane Raschke, Kurt Zoglauer, Andrea Rupps

**Affiliations:** ^1^Department of Plant Systematics and Evolution, Institute of Biology, Humboldt-Universität zu Berlin, Berlin, Germany; ^2^BioForBois, Wood & Construction Industry Department, FCBA, Cestas, France

**Keywords:** homologous genes, homologous and heterologous overexpression, genetic transformation, *WOX2*, WUSCHEL, LEC1

## Abstract

Although full sequence data of several embryogenesis-related genes are available in conifers, their functions are still poorly understood. In this study, we focused on the transcription factor *WUSCHEL-related HOMEOBOX 2 (WOX2)*, which is involved in determination of the apical domain during early embryogenesis, and is required for initiation of the stem cell program in the embryogenic shoot meristem of *Arabidopsis*. We studied the effects of constitutive overexpression of *Pinus pinaster WOX2* (*PpWOX2*) by *Agrobacterium*-mediated transformation of *P. pinaster* somatic embryos and *Arabidopsis* seedlings. Overexpression of *PpWOX2* during proliferation and maturation of somatic embryos of *P. pinaster* led to alterations in the quantity and quality of cotyledonary embryos. In addition, transgenic somatic seedlings of *P. pinaster* showed non-embryogenic callus formation in the region of roots and subsequently inhibited root growth. Overexpression of *PpWOX2* in *Arabidopsis* promoted somatic embryogenesis and organogenesis in a part of the transgenic seedlings of the first and second generations. A concomitant increased expression of endogenous embryogenesis-related genes such as *AtLEC1* was detected in transgenic plants of the first generation. Various plant phenotypes observed from single overexpressing transgenic lines of the second generation suggest some significant interactions between *PpWOX2* and *AtWOX2*. As an explanation, functional redundancy in the WOX family is suggested for seed plants. Our results demonstrate that the constitutive high expression of *PpWOX2* in *Arabidopsis* and *P. pinaster* affected embryogenesis-related traits. These findings further support some evolutionary conserved roles of this gene in embryo development of seed plants and have practical implications toward somatic embryogenesis induction in conifers.

## Introduction

With respect to the divergence of angiosperms and gymnosperms, many morphological differences of embryogenesis and embryo development have to be considered (Bowe and Coat, 2000; Cairney et al., 2006; Cairney and Pullman, 2007). Many studies using *Arabidopsis* as a model plant have increased our knowledge of the function of embryogenesis-related genes in angiosperms (Laux et al., 1996; Lotan et al., 1998; Boutilier et al., 2002; Haecker et al., 2004; Breuninger et al., 2008; Jeong et al., 2011; Chung et al., 2016; Horstman et al., 2017). In contrast, little is known about the function of genes that regulate embryogenesis in conifers (Cairney and Pullman, 2007; De Silva et al., 2008; Trontin et al., 2016a). This is because of particular challenges of conifers. These species have long breeding cycles, large physical size, and slow growth. As a result, a number of powerful genetic approaches used in model plants, such as identification of zygotic embryo-defective mutants and T-DNA insertional mutagenesis, are impracticable. Moreover, recalcitrance to vegetative propagation through conventional or tissue culture methods (mostly aging/phase-change effects) often results in lack of an efficient system for direct plant regeneration from selected superior trees. Available methods are, therefore, mostly “retroactive” and based on somatic embryogenesis initiation from immature seeds coupled with cryopreservation to preserve the juvenility of embryogenic tissue. Selected genotypes after field evaluation of somatic seedlings in clonal tests can be propagated from a cryopreserved juvenile stock. This is currently the preferred and powerful strategy in conifers to overcome recalcitrance of mature trees (Klimaszewska and Cyr, 2002; Bonga et al., 2010) to achieve clonal propagation of superior genotypes and for gene functional studies by genetic transformation of embryogenic cultures (Klimaszewska et al., 2007, 2009, 2016).

The first step for assignment of gene functions in conifers is usually based on sequence similarities between conifers and model angiosperms. Most embryogenesis-related genes identified in *Arabidopsis* (a global network of ca. 300–450 genes) (Tzafrir et al., 2004; De Smet et al., 2010) have homologous candidate sequences in conifers (Cairney and Pullman, 2007; Zhang et al., 2012). The second one is analysis of transgenic plants following overexpression and/or knock-down of the candidate genes. Such reverse genetics approach for functional gene studies remains challenging in most conifers, because significant genomic resources are needed (genome sequence, expressed sequence tags, etc.), as well as efficient genetic transformation and plant regeneration systems (Trontin et al., 2007). Significant progress has been achieved in several conifer species of high economical interest (*Pinus*, *Picea*, and *Larix*) based on *Agrobacterium*-mediated genetic transformation of embryogenic cultures and plant regeneration by somatic embryogenesis (Trontin et al., 2007; Klimaszewska et al., 2009, 2016; Bonga et al., 2010). Such a system has been developed for reverse genetics in maritime pine (*Pinus pinaster* Ait.) during the past 15 years (Trontin et al., 2002, 2007, 2016b; Lelu-Walter et al., 2016) and was implemented by multinational EU consortia (e.g., Sustainpine, 2010–2013^1^; ProCoGen, 2012–2015^2^) for functional gene studies (Trontin et al., 2013; El-Azaz et al., 2020).

**FIGURE 1:**
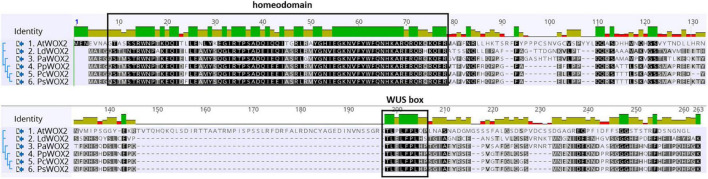
Comparative WOX2 amino acid sequences in the angiosperm *Arabidopsis thaliana* (AtWOX2) and in 5 conifers, namely, *Larix decidua* (LdWOX2), *Picea abies* (PaWOX2), *Pinus pinaster* (PpWOX2), *Pinus contorta* (PcWOX2), and *Pinus sylvestris* (PsWOX2). Introduced gaps in the amino acid sequences are indicated by dashes. Boxes show the putative WOX homeodomain and WUS box. Amino acid identity is shown by a color code.

An embryogenesis-related gene that is crucial for early embryo development in angiosperms is *WUSCHEL-related HOMEOBOX 2* (*WOX2*). In *Arabidopsis*, *WOX2* is required for cell fate decisions and domain delineation in the apical domain during embryo development, including initiation of shoot meristem stem cells (Haecker et al., 2004; Xiao et al., 2006; Breuninger et al., 2008; Zhang et al., 2017). In post-embryonic development, *WOX2* seems to be involved in lateral organ formation and separation (Chung et al., 2016). The *WOX2* gene encodes a member of the *WOX* family of homeodomain transcription factors. WOX proteins share a homeobox that encodes a WUS-type homeodomain. In addition to the homeodomain, many WUS/WOX proteins contain a conserved WUS-box (TLPLFPMH) located downstream of the homeodomain in both angiosperms and gymnosperms (Haecker et al., 2004; Nardmann and Werr, 2006; see also Figure 1). Dolzblasz et al. (2016) reported that the canonical WUS-box is essential for stem cell maintenance within shoot apical meristem in *Arabidopsis*. Moreover, several overexpression studies indicate that WOX family members containing the conserved WUS-box are involved in plant growth and development. For example, induced overexpression of *WOX5* in *Arabidopsis* produces extra layers of stem cells in columella root cap (Zhang et al., 2010). Overexpression of *WOX1* in *Arabidopsis* leads to defects in meristem development (Zhang et al., 2011). Transgenic plants overexpressing *WUSCHEL* (*WUS*, founding member of the *WOX* family) in *Arabidopsis* led to high frequency of somatic embryo (SE) formation in all tissues without any need for exogenous plant hormone supply (Laux et al., 1996; Zuo et al., 2002). Furthermore, in angiosperms other than *Arabidopsis*, the overexpression of *AtWUS* induced somatic embryogenesis and callus formation (Arroyo-Herrera et al., 2008; Solís-Ramos et al., 2009; Bouchabke-Coussa et al., 2013).

In conifers, the transcription factor *WOX2* is considered as an important developmental regulator during early somatic embryogenesis (Klimaszewska et al., 2010; Trontin et al., 2016a) and has been proposed as a putative marker for effective initiation of somatic embryogenesis and to predict embryogenic potential (Palovaara and Hakman, 2008; Klimaszewska et al., 2010, 2011; Miguel et al., 2016; Alvarez et al., 2018). Zhu et al. (2016) revealed that the *WOX2* from *Picea abies* (*PaWOX2*) plays more crucial roles during early embryogenesis (i.e., protoderm formation and suspensor expansion) than during late embryogenesis.

Genomic resources are now accumulating in model conifers, especially for *Picea* and *Pinus* species (Birol et al., 2013; Nystedt et al., 2013; Neale et al., 2014; Plomion et al., 2016). For this study, we focused on *P. pinaster*, as the production of similar genomic sequence data was initiated during the European ProCoGen project (2011–2015^2^). Moreover, a reference transcriptome has been made available for this species (SustainpineDB database^1^) (Canales et al., 2014).

To our knowledge, there has been no report up to now on the overexpression of *WOX2* isolated from conifers in both gymnosperms and, more specifically, angiosperms. The genetic transformation of *Pinus* is a long and difficult process typically achieved in about 12 months. Deregulating *WOX2* with same binary vector in both *P. pinaster* and *Arabidopsis* would provide additional insights into evolutionary conserved roles of this gene in gymnosperms and angiosperms, particularly during somatic embryogenesis. In this study, we analyzed the constitutive overexpression of *WOX2* isolated from *P. pinaster* (*PpWOX2*) in both *P. pinaster* SE and *Arabidopsis* seedlings. Our results showed that the endogenous expression of *PpWOX2* is enhanced in early proliferating embryos and significantly decreased during SE development in *P. pinaster*. Constitutive overexpression of *PpWOX2* in *P. pinaster* embryogenic cultures affected SE development and promoted callus formation in the root region of regenerated somatic seedling. In *Arabidopsis* seedlings, *PpWOX2* overexpression led to increased propensity to somatic embryogenesis and organogenesis, likely by alteration of expression patterns of embryogenesis-related genes.

## Materials and Methods

### Plant Materials and Growth Conditions

#### P. pinaster

Somatic embryos (SEs) of a *P. pinaster* embryogenic line (PN519) were used for genetic transformation and transgenic plant production. PN519 was initiated at FCBA in 1999 from an immature zygotic embryo (full-sib progeny, Breton et al., 2006) according to the method of Bercetche and Pâques (1995). This line has good abilities for both genetic transformation and plant regeneration by somatic embryogenesis and has been, therefore, extensively characterized for the past 15 years (Trontin et al., 2002, 2007, 2013, 2016b; Breton et al., 2006; Lelu-Walter et al., 2016).

#### A. thaliana

Seeds of *Arabidopsis thaliana* (L.) Heynh. (ecotype Columbia-0) were obtained from the Nottingham *Arabidopsis* Stock Center^3^. For germination, the *Arabidopsis* seeds were soaked first in tap water at 4°C for 2 days and thereafter transferred to potting soil (Stender, Schermbeck, Germany) and grown for 4–6 weeks at 21–23°C under short day conditions (10-h light/14-h dark cycle) with illumination at ∼120 μE m^–2^ s^–1^ provided by HQI Powerstar 400W/D (Osram Licht AG, Munich, Germany). For flower induction, plants were cultivated under the same but longer day conditions (16-h light).

Both SEs and non-embryogenic calluses were obtained from wild-type (WT) *Arabidopsis* plant seedlings after induction following the method reported by Gaj (2001). WT SE and non-embryogenic WT calluses were used as positive (embryogenic) and negative (non-embryogenic) controls, respectively.

#### Characterization of WOX2 in P. pinaster (PpWOX2) and Cloning

*In silico* search for *WOX2* sequences in the *P. pinaster* expressional database (see Canales et al., 2014) was performed by Philippe Label (INRAE, France). The cDNA sequence of a putative *P. pinaster* homolog to *WOX2* (*PpWOX2*) was identified in SustainpineDB (nine unigenes assigned to conifer *WOX2*) and submitted under accession number (Acc.) KY773924.1 at the National Center for Biotechnology Information (NCBI) GenBank, and its cloning was performed. *PpWOX2* was concurrently sequenced by Alvarez et al. (2018) and deposited in GenBank under accession number KU962991. The two published sequences are identical (570 bp).

Alignment of the deduced amino acid sequence of *PpWOX2* [Acc. ARS01278 in this study; concurrently deposited by Alvarez et al. (2018), Acc. ANC94872] with homologous WOX2 protein sequences from *Pinus sylvestris* (PsWOX2, Acc. CAT02937.2; Nardmann et al., unpublished), *Pinus contorta* (PcWOX2, Acc. ADR10436.1; Park et al., 2010), *Picea abies* (PaWOX2, Acc. CAL18267.1; Palovaara and Hakman, 2008), *Larix decidua* (LdWOX2, Acc. AEF56564.2; Rupps et al., 2016), and *A. thaliana* (AtWOX2, Acc. NP_200742.2; Tabata et al., 2000) was carried out using the Geneious R10.2.3 software (Biomatters Ltd., Auckland, New Zealand). Gaps were included in the sequences to optimize the alignment.

#### Binary Vector Construction for Constitutive Overexpression of PpWOX2

To generate the *35S::PpWOX2* vector, the *GUSPlus* gene of the *35S::GUSPlus* cassette from the binary vector *pCAMBIA1305.2* (GenBank Acc. AF354046)^4^ was replaced with the *PpWOX2* gene isolated from *P. pinaster* (Acc. KY773924). The *GUSPlus* gene was excised from *pCAMBIA1305.2* by restriction with *Nco*I and *Pml*I. The entire open reading frame of *PpWOX2* was amplified from cDNA (obtained from total RNA extract) using specific primers (Table 1). The PCR fragment (570 bp) was then ligated into the *Nco*I and *Pml*I sites of the digested *pCAMBIA1305.2*. The resulting *35S::PpWOX2* binary vector was checked by sequencing and introduced into the disarmed *Agrobacterium tumefaciens* strain *C58/C1* using the freeze-thaw method (Nishiguchi et al., 1987). The *pCAMBIA1305.2* vector harbors a constitutive expression cassette (*35S::HPTII*) for selection of transgenic events with hygromycin. This antibiotic is effective in maritime pine (Trontin et al., 2002, 2007).

**TABLE 1:** List of gene-specific primers used for transgene analyses and reverse transcription quantitative (RT-q) PCR in *Pinus pinaster* and *Arabidopsis.*

Gene	Oligonucleotide name	Oligonucleotide sequence (5′ – 3′)
**Primers for transgene analyses**		
*HPTII*	HPTII-fw	AAC ATC GCC TCG CTC CAG TCA ATG A
	HPTII-rev	AAT AGC TGC GCC GAT GGT TTC TAC A
*NPTII*	NPTII-fw	CTA TGG CTG GAA GGA AAG CTG
	NPTII-rev	TCA GGC TTG ATC CCC AGT AAG
*PpWOX2*	PpWOX2-fw	TGG AGG CCA TGT ACA GTC AA
	PpWOX2-rev	GCC AGG TGG TTG ATG AAA CT
*CaMV35S*	CaMV35S	CTA TCC TTC GCA AGA CCC TTC
	T35S_rev	GGT CAC TGG ATT TTG GTT TTA GG
**Primers for RT-qPCR in *P. pinaster* (reference genes)**		
*Helicase*	PpHELI_fw	GGA GCT CTT GAT GAG ATG GAG
	PpHELI_rev	GGA AGG ACA AAT GGA TCA CG
*Expressed protein At2g32170*	Pp32170_fw	TCT TTA CTC CCA TGG CGT TC
	Pp32170_rev	TGT TTG GGA GAT TGC TGA AAG
*Mitosis Protein YLS8*	PpYLS8_fw	GTG GAT CAG GCC ATT CTA GC
	PpYLS8_rev	ACC TCC GTT ATG TCC ACC AG
**Primers for RT-qPCR in *Arabidopsis* (reference genes)**		
*SAND*	SAND-fw	AAC TCT ATG CAG CAT TTG ATC CAC T
	SAND-rev	TGA TTG CAT ATC TTT ATC GCC ATC
*PP2A*	PP2A-fw	AAG GTA AAG AAG ACA GCA ACG A
	PP2A-rev	GCC AAC ATT AAC ATT AGT AGC AGA G
*PEX4*	PEX4-fw	CCT CTT AAC TGC GAC TCA GG
	PEX4-rev	TTT GTG CCA TTG AAT TGA ACC C
**Primers for RT-qPCR in *P. pinaster* and/or *Arabidopsis* (genes of interest)**		
*PpWOX2*	3Pinpi_WOX2_ORF_fw	ATC CGG CAT CGC TGA ATA C
	3Pinpi_WOX2_ORF_rev	TAC TTG CCA GGA TGC TGA GG
*AtLEC1*	At-LEC-ORF-B-fw	CAT GGT TAT GGG AGG TGG TC
	At-LEC-ORF-B-rev	AGA GCC ACC ACC AAC ACT G
*AtWUS*	At-WUS-ORF-fw	ACC CAA CTC GGT TAT GAT GG
	At-WUS-ORF-rev	GCT GGG ATA TGG CTT GTT ATG
*AtWOX2*	At-WOX2-ORF-fw	TCA AAC GTG GGT TGT GTC AG
	At-WOX2-ORF-rev	AGC CAC CAC TTG GAA TCA TC

#### Agrobacterium-Mediated Transformation of P. pinaster

*Agrobacterium*-mediated genetic transformation of *P. pinaster* proliferating SE was performed with proliferating SE, 7 days after sub-culture, i.e., during the phase of active SE growth on proliferation medium.

The transformation procedure was a combination of a reference FCBA/INRAE protocol designed during the Sustainpine project for *P. pinaster* [Trontin and Lelu-Walter, 2010; partially published in Trontin et al. (2002, 2007)] and the “droplet method” adapted for *Larix decidua* (Taryono, 2000). The co-cultivation procedure was performed according to the “droplet method”. One colony of *Agrobacterium C58/C1* was inoculated in a liquid YEB medium with appropriate antibiotics at 28°C for approximately 16 h while shaking (200 rpm). The optical density at 600 nm (OD_600_) of *Agrobacterium* suspension was adjusted to about 0.5. Each clump of immature proliferating SEs was inoculated with one droplet (30 μl) of the *Agrobacterium* suspension. The SEs and agrobacteria were co-cultivated for 2 days in an MSG medium (Klimaszewska, 1989) with 0.1 M acetosyringone in darkness at 21–23°C. For elimination of agrobacteria, the co-cultivated SEs were washed with a 200-ml liquid MSG medium supplemented with 300 mg l^–1^ timentin (MSG-T) for 30 min. Afterward, the SEs were collected on filter paper in a Büchner funnel using a vacuum pump and a short low-pressure pulse. The filter paper was subsequently placed into a solidified MSG-T medium for 10 days. Selection for transgenic embryo clumps (transgenic lines) was performed on an MSG-T medium supplemented with 10 mg l^–1^ hygromycin for 2 weeks. The SEs were then sub-cultured every 2 weeks into the MSG-T medium plus 15 mg l^–1^ hygromycin for 6 weeks. After the selection procedure, transformation efficiency was calculated as the number of hygromycin-resistant lines confirmed by PCR analysis (PCR+ lines) per gram fresh mass (FM) of co-cultivated proliferating SEs.

All transformation experiments were performed twice, and up to 10 PCR+ lines were cryopreserved.

After preliminary SE maturation experiments, we selected two independent PCR+ lines that showed quite stable *PpWOX2* overexpression during SE proliferation (lines OE_#11 and OE_#15). The maturation experiments were performed at the same time for both *35S::PpWOX2* lines, as well as for one empty vector transgenic control line, *pCAMBIA 1305.2* (EV-pC05), and the non-transgenic WT PN519 control line. All transgenic and control lines were managed simultaneously and at the same subculture frequency to avoid any differences related with aging effect.

#### Culture of Embryogenic Line, Somatic Embryos, and Seedlings of *P. pinaster*

**FIGURE 2:**
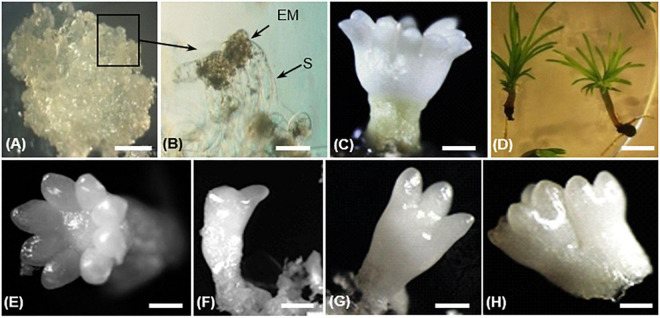
Different steps of somatic embryo (SE) development in *Pinus pinaster* **(A–D)** and morphology of harvested cotyledonary SEs **(E–H)** in transgenic *35S::PpWOX2* lines and control lines (transgenic EV-pC05 line and the non-transgenic WT).**(A)** Embryogenic culture containing proliferating immature SE; **(B)** proliferating SEs showing embryo proper (EP) and suspensor cells (S); **(C)** cotyledonary SE after 14 weeks in maturation medium; **(D)** somatic seedlings after 8 weeks in conversion medium. Cotyledonary SEs from *PpWOX2* and control lines were classified according to different categories such as **(E)** normal (cotyledons with symmetrical radial development), **(F,G)** deformed (cotyledons with asymmetrical development), and **(H)** not separated, fused embryos. Scale bars: **(A)** 3 mm, **(B)** 0.1 mm, **(B,C,E–H)** 2 mm, and **(D)** 5 mm.

The whole process of SE development since reactivation from the cryopreserved stock involved three developmental stages: proliferation, maturation, and conversion (Ramarosandratana et al., 2001). In proliferating embryogenic tissues, an immature SE consists of an embryonic region of small, densely cytoplasmic cells subtended by long, highly vacuolated suspensor cells (Figures 2A,B). During maturation, the embryonic region became more prominent and opaque. Finally, a cotyledonary SE shows a shoot apex and a root pole containing primary meristems, a well-defined cotyledon ring, and a hypocotyl (Figure 2C). Cotyledonary SEs have a potential to germinate and develop into rooted somatic seedlings (Figure 2D).

#### Maintenance and Cryopreservation of Proliferating Immature Somatic Embryos

The PN519 embryogenic line was initially reactivated from the FCBA cryopreserved stock following the method described by Harvengt (2005) but using a modified Litvay et al. (1985) basal formulation (mLV) as reported in Klimaszewska et al. (2001, 2007). Proliferating immature SEs were sub-cultured every 10–14 days on Petri dishes in an mLV basal medium (Klimaszewska et al., 2001, 2007) supplemented with 2 μM 2,4-D (2,4-dichlorophenoxyacetic) acid (Sigma-Aldrich, Germany) and 1 μM BAP (6-benzylaminopurine; Sigma-Aldrich, Germany). Embryogenic cultures were maintained in darkness at 22 ± 2°C. Re-cryopreservation of PN519 transgenic and non-transgenic control WT lines was performed using a protocol previously established for *Abies* and *Larix* species (Zoglauer et al., 2003). Any thawed SE culture batch was reactivated within 1–2 months prior to maturation.

#### Somatic Embryo Maturation, Yield in Cotyledonary Somatic Embryos, and Morphological Analysis

Development of SEs to reach the cotyledonary stage was performed on mLV-based maturation medium as the best option reported for maritime pine (Lelu-Walter et al., 2016; Trontin et al., 2016b). The method described by Morel et al. (2014) was used with slight modifications. Briefly, 1 g of proliferating SEs (3–5 days after sub-culture in mLV proliferation medium) was washed first and then suspended in 10 ml of the mLV liquid medium without plant growth regulators (PGRs). An aliquot of a suspension (1 ml containing ∼100 mg proliferating SEs) was spread on a sterile filter paper (Whatman No. 2, diameter 11 cm), air-dried at ambient temperature, and placed on a Petri dish in an mLV medium supplemented with 80 μM abscisic acid (ABA; Duchefa, Haarlem, The Netherlands) and 0.2 M sucrose, and solidified with 1% (w/v) gellan gum (Gelrite; Duchefa, Haarlem, The Netherlands). All maturation cultures were maintained in darkness and at 22 ± 2°C. Each maturation experiment was performed on 10 Petri dishes and repeated 2–3 times.

After 12–14 weeks, SE development reached the cotyledonary stage (Morel et al., 2014). Embryogenic potential was estimated as the number of cotyledonary SEs per g FM of embryogenic tissue. Cotyledonary SEs of *PpWOX2* transgenic lines and controls (transgenic EV-pC05 line and non-transgenic WT) were classified according to their morphological characteristics as “normal” (cotyledons with symmetrical radial development, Figure 2E), “deformed” (cotyledons with asymmetric development, e.g., one cotyledon, two cotyledons, etc., Figures 2F,G), or “not separated,” fused embryos (two embryos not clearly separated from each other, Figure 2H; Marum et al., 2009).

#### Conversion of Cotyledonary Somatic Embryos Into Somatic Seedlings, and Acclimatization

To convert cotyledonary SEs into plantlets, fully developed embryos with well-shaped cotyledons (normal SEs) were placed horizontally onto Petri dishes on the surface of an mLV medium supplemented with 3% sucrose (w/v) and solidified with 0.4% (w/v) Gelrite and 0.06% (w/v) plant agar (Duchefa, Haarlem, The Netherlands). The cultures were maintained in darkness at 22 ± 2°C for 10 days. Afterward, the seedlings were transferred to a long day photoperiod (14-h light, 120 μE m^–2^ s^–1^, OSRAM Lumilux Cool White light) at 22 ± 2°C for 6–8 more weeks. Seven embryos were placed in each plate. Three replicates were used for each treatment, and experiments for SE conversion were repeated three times. After 6 weeks, the SEs were scored as converted into viable somatic seedlings if an elongated epicotyl, green cotyledons, and primary needles as well as an elongating root apex were observed. The percentage of somatic seedlings with root elongation was determined after 8 weeks of culture.

#### Callus Induction From Transgenic *PpWOX2* Somatic Seedlings in *P. pinaster*

The ability of different explants from *P. pinaster* somatic seedlings to initiate embryogenic tissue and/or non-embryogenic callus was tested for *PpWOX2* transgenic lines, the empty vector (EV-pC05), and non-transgenic WT control lines. Needle/hypocotyl and root explants of somatic seedlings were collected 6–8 weeks after germination of cotyledonary SEs and cultivated in a conversion medium, i.e., mLV basal medium (Klimaszewska et al., 2001, 2007) without PGRs. The cultures were incubated in darkness for 8 weeks at 21–23°C.

#### *Agrobacterium*-Mediated Transformation of *A. thaliana*

*Agrobacterium*-mediated transformation of *Arabidopsis* seedling plants using the 35S::*PpWOX2* vector was performed with a modified floral-dip method to obtain T_0_ seeds (Weigel and Glazebrook, 2002). To select transgenic lines and for regeneration of first-generation (T_1_) plants, water-soaked and cold-treated (2 days at 4°C) T_0_ seeds were surface-sterilized by immersion in 1.5% NaOCl (active chlorine) for 15 min, followed by three rinses in autoclaved *Aqua purificata* (aqua pur.). Then, the T_0_ seeds were cultured *in vitro* in an MS medium (Murashige and Skoog, 1962) supplemented with 20 mg l^–1^ hygromycin and incubated for 4–6 weeks under short day conditions (10-h light, 120 μE m^–2^ s^–1^). Hygromycin-resistant transgenic T_1_ plants obtained from treated T0 seeds were transferred to potting soil for 3–4 weeks under long day conditions to promote flowering. Plants from two independent T_1_ transgenic lines (PpWOX2-line #1 and PpWOX2-line #2) were self-pollinated. Resulting seeds of the T_1_ generation were harvested and subsequently cultured in an MS medium supplemented with 20 mg l^–1^ hygromycin to regenerate T_2_ transgenic plants. The two selected T_1_ transgenic lines were shown to be homozygotes for the *PpWOX2* transgene based on both hygromycin-resistance assay and PCR analysis of their T_2_ progenies (data not shown).

#### Histological Studies on *Arabidopsis* Tissues

For preparation of histological sections, *Arabidopsis* tissues (green callus containing embryo-like or leaf-like structures) were fixed in 3% (v/v) glutaraldehyde for 24 h at 4°C. Samples were rinsed in 1 × PBS and dehydrated in an ethanol series (30, 50, 70, 90, and 100%) at 4°C. The samples were then infiltrated in an ethanol:historesin solution (glycol methacrylate, GMA-Leica) ratio series (4:1, 4:2, 4:3, 4:4, 3:4, 2:4, 1:4, and finally 100% historesin) and embedded in historesin (15 ml infiltration solution + 1 ml hardener; Leica, Wetzlar, Germany). Five-μm thick slices were obtained with a rotary microtome (HM 355S; Microm, Walldorf, Germany). The sections were floated on aqua pur. at room temperature, picked up in clean glass slides, and air-dried at 42°C. The sections were stained with 0.5% (w/v) ponceau S for 6 min, rinsed in aqua pur., and stained again with 0.5% (w/v) methylene blue for 15 s followed by rapid rinse in aqua pur. The stained sections were examined using an Olympus SZ X12 microscope.

### Molecular Analyses

#### Confirmation of Transgene Integration

Plant genomic DNA from both *P. pinaster* and *Arabidopsis* materials (transgenic line, embryo, and seedlings) was isolated from a 100-mg FM tissue according to a modified Doyle (1990) protocol (Dumolin et al., 1995).

To confirm genetic transformation, putative independent transgenic events (hygromycin-resistant lines) were tested by PCR using specific primers (Table 1) targeting *PpWOX2* (*PpWOX2-*fw and T35S_rev, 592-bp fragment; *PpWOX2-*rev and CaMV35S, 315-bp fragment) and *HPTII (HPTII-fw, HPTII-rev*, 412-bp fragment).

For detection of persistent *Agrobacterium* in the putative transgenic events (false positive amplification), PCR was also performed with *NPTII*-specific primers (Table 1, 557-bp fragment).

#### Gene Expression Analysis by Reverse Transcription-Quantitative PCR

Total RNA from proliferating SEs and cotyledonary SEs of *P. pinaster* was isolated with RNeasy^®^ Plant Mini Kit (Qiagen, Venlo, The Netherlands) using an RLC lysis buffer containing guanidine hydrochloride according to manufacturer’s instructions. Total RNA from somatic seedlings of *P. pinaster* (germinated for 6–8 weeks) was isolated according to Chang et al. (1993). Total RNA from *Arabidopsis* seedlings was extracted using TRIzol (Invitrogen) following manufacturer’s instructions. cDNA was synthesized from 1 μg total RNA using QuantiTect^®^ Reverse Transcription Kit (Qiagen, Venlo, The Netherlands) as described in the manufacturer’s instructions.

Reverse transcription-quantitative (RT-q) PCR reactions were performed with a CFX96™ Real-Time PCR detection system (Bio-Rad, Hercules, CA, United States). Reactions were performed with 1 μl template (10 ng cDNA), 5 μl 2 × SYBR Green Master mix (SensiMix™ SYBR No-ROX Kit; Bioline, Memphis, TN, USA), 0.3 μl forward and reverse primers (10 μmol μl^–1^), and aqua pur. to a final volume of 10 μl.

Measurements were carried out on three reactions per cDNA sample (technical replicates) and three cDNA samples that were synthesized from independent RNA samples (biological replicates). Negative controls, i.e., either autoclaved aqua pur. or RNA without reverse transcriptase as template, were included in each run. Three reference genes encoding Helicase (TC151784 from *P. pinaster)*, expressed protein At2g32170 (TC125990 from *Pinus taeda*), and mitosis protein YLS8 (TC136697 from *P. pinaster*) were used in combination for normalization of the relative expression of *PpWOX2* in *P. pinaster* (Table 1). The expression of these genes was shown to be constant during SE development (Arndt, 2013). The relative expression of *PpWOX2* (both transgene and endogenous genes), *AtWOX2*, *AtWUSCHEL* (*AtWUS*), and *AtLEC1* in *Arabidopsis* was normalized to the expression levels of three reference genes from *Arabidopsis* (*SAND:* At2g28390, *PP2A:* At1g13320, and *PEX4:* At5g25760, Table 1) validated by Czechowski et al. (2005).

Real-time RT-qPCR data were analyzed using Bio-Rad CFX Manager Version 1.6.541.1028 and qbase PLUS. Accurate normalization based on multiple internal control genes was achieved following the method of Vandesompele et al. (2002).

## Results

### Identification of *PpWOX2* and Expression During Somatic Embryo Development in *P. pinaster*

The cDNA sequence (570 bp) of a putative *P. pinaster* homolog to *WOX2* (*PpWOX2*) was independently identified in SustainpineDB [(Acc. KY773924); in this study: Acc. KU962991, Alvarez et al., 2018].

The deduced PpWOX2 protein sequence (Acc. ARS01278; in this work: Acc. ANC94872, Alvarez et al., 2018) shares conserved characteristic features of the plant WOX family (Alvarez et al., 2018). In addition to the conserved homeodomain, many plant WUS/WOX proteins contain a conserved WUS-box (TLPLFP) located downstream of the homeodomain (Figure 1, Haecker et al., 2004; Nardmann and Werr, 2006), including in *Pinus pinaster* (Alvarez et al., 2018). Such a putative WUS-box was identified in the PpWOX2 protein sequence and shows the same differences in amino acid sequence compared to *Arabidopsis* (TLPLFPLQP) as other conifers from genera *Pinus*, *Picea* and *Larix* (TLELFPLHP).

**FIGURE 3:**
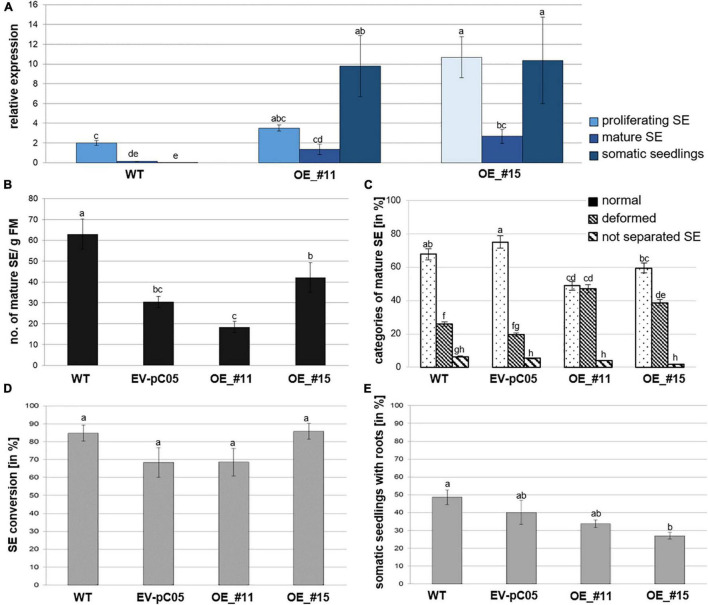
Analysis of *35S::PpWOX2* overexpressing *P. pinaster* lines #11 and #15 (OE_#11 and OE_#15) and controls (WT, EV-pC05) with regard to *PpWOX2* transgene expression following **(A)** RT-qPCR analysis, **(B)** SE maturation, **(C)** cotyledonary SE morphology, and **(D)** conversion **(D)** as well as **(E)** root development. **(A)** Relative expression of *PpWOX2* in proliferating SEs, cotyledonary SEs, and somatic seedlings by RT-qPCR. **(B)** Number of cotyledonary SEs per g FM after 12–14 weeks in maturation medium. **(C)** Percentage of cotyledonary SEs (*n* = 70) with different morphological phenotypes classified as normal, deformed, or not separated (fused) after 12–14 weeks in maturation medium. **(D)** Percentage of SEs (*n* = 21) converted into somatic seedlings after 6 weeks in germination medium. **(E)** Percentage of somatic seedlings (*n* = 21) with active root growth after 8 weeks in conversion medium. Embryos of control transgenic line EV-pC05 were transformed with *pCAMBIA1305.2* (empty vector). WT line was the original PN519 line used for transformation experiment. Each value represents mean ± standard error obtained from three biological replicates and **(A)** 3, **(D,E)** 4, or **(B,C)** 7–10 technical replicates. Bars with different letters are significantly different (ANOVA, *P* < 0.05).

*Pinus pinaster WOX2* expression (both transgene and endogenous genes) was investigated during normal development of *P. pinaster* SE by RT-qPCR (wild-type samples). Significant variation was detected by ANOVA [*F*(8,18) = 23.479, *P* < 0.05; Figure 3A]. Relative expression was highest in proliferating immature SEs and then significantly decreased in cotyledonary SEs. A very low level of *PpWOX2* expression was detected in 6–8-week–old somatic seedlings although not significantly different from that of cotyledonary SEs.

### Transformation Efficiency of *P. pinaster* PN519 Line and *PpWOX2* Expression in Transgenic Lines

**FIGURE 4:**
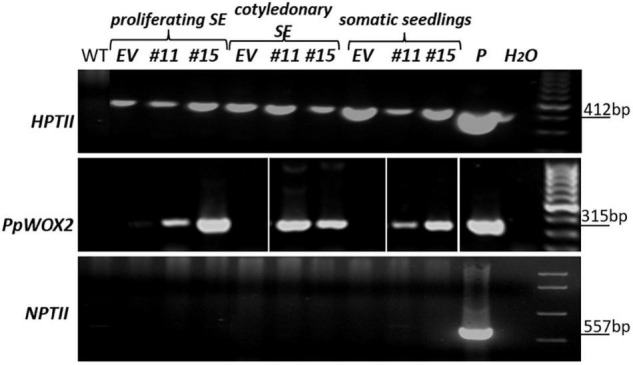
PCR analyses to confirm the presence and stability of the transgene in *35S::PpWOX2 P. pinaster* lines in various developmental steps from proliferating immature SEs to cotyledonary SEs and somatic seedlings. Lines #11 and #15 are PCR-positive (PCR+) throughout SE development. PCR amplification of *HPTII* (412 bp), *PpWOX2* (315 bp), and *NPTII* (557 bp). Empty vector (EV): embryos of control line EV-pC05 transformed with *pCAMBIA1305.2*, *P* = *35S::PpWOX2-*isolated plasmid (positive PCR control). WT, wild-type, non-transgenic PN519 control line. H_2_O, negative PCR control (water as template).

The transformation efficiency of proliferating SEs with the *35S::PpWOX2* construct or the control empty vector *pCAMBIA1305.2* was 11.9 and 26.0 PCR+ lines g^–1^ FM, respectively. The presence of T-DNA (*HPTII* and *PpWOX2* PCR detection) was confirmed in hygromycin-resistant *35S::PpWOX2* lines in different developmental steps (proliferating SEs, cotyledonary SEs, and somatic seedlings; Figure 4). The *NPTII* bacterial selectable marker was not detected in these PCR+ lines during SE development (Figure 4), supporting that PCR+ lines are free from contaminating agrobacteria. Ten 35S::*PpWOX2* PCR+ lines and five transgenic control lines (EV-pC05) could be cryopreserved.

Following RT-qPCR, two independent *35S::PpWOX2* lines (#11 and #15) showed higher *PpWOX2* relative expression compared to the wild type (WT) in the three analyzed developmental steps [*F*(8,23) = 7.27, *P* < 0.05; Figure 3A]. Observed differences were only significant for line #15 in the proliferating and mature stages but highly significant for both transgenic lines (#11 and #15) in the seedling stage. Similar results were obtained for any of the eight additional *35S::PpWOX2* lines that were generated and cryopreserved (data not shown). Similar to WT, relative expression was decreased in cotyledonary SEs compared to proliferating SEs (although only significant for line #15), suggesting some phase change effect on transition between early and late embryogenesis. Although some differences may exist, in our experiments, *PpWOX2* expression was similar between proliferating SEs and somatic seedlings (no significant differences) in both lines #11 and #15.

### Development of *P. pinaster* Somatic Embryos Overexpressing *PpWOX2*

The effect of high constitutive overexpression of *PpWOX2* on SE development in *P. pinaster* was analyzed by evaluating SE maturation and conversion into plantlets for the *35S::PpWOX2* lines (#11 and #15) and controls (WT and EV-pC05).

*PpWOX2* over-expressing lines #11 and #15 produced similar and significantly reduced yields in cotyledonary SEs compared to WT (Figure 3B). However, embryos of the control transgenic line EV-pC05 showed a similar significant trend [*F*(4,84) = 17.046, *P* < 0.05; Figure 3B], suggesting some technical issue related to the genetic transformation procedure (see Discussion and Supplementary Table 1). In maritime pine, it has been reported that hygromycin selection could result in reduced yields in cotyledonary SEs (Trontin et al., 2007).

No significant morphological differences were observed between WT and transgenic control line EV-pC05. In contrast, the ANOVA revealed significant variation in normal and deformed embryos among *PpWOX2* transgenic and control lines [*F*(11,213) = 30.16, *P* < 0.05; Figure 3C]. The transgenic lines showed significantly reduced yields in normal embryos. Accordingly, a significant, higher percentage of proliferating SEs from lines #11 (47%) and #15 (39%) developed into deformed embryos, mostly with asymmetric development of cotyledons when compared to the WT (25%) and control line EV-pC05 (18%).

The overexpression of *PpWOX2* had no detectable early effect on conversion of cotyledonary SEs into somatic seedlings for both *35S::PpWOX2* lines #11 and #15 as compared with the WT and EV-pC05 (Figure 3D). High conversion rates were observed for all lines (68–85%). Accordingly, no significant difference in the frequency of somatic seedlings showing active root growth could be detected between *35S::PpWOX2* lines and the EV-pC05 control (Figure 3E). As some reduced frequency is observed for all transgenic lines compared to the WT, especially in the case of line #15 *F*(4,19) = 5.002, *P* = 0.006; Figure 3E, (as for SE yield), some adverse effects of the genetic transformation procedure have again been suggested.

**FIGURE 5:**
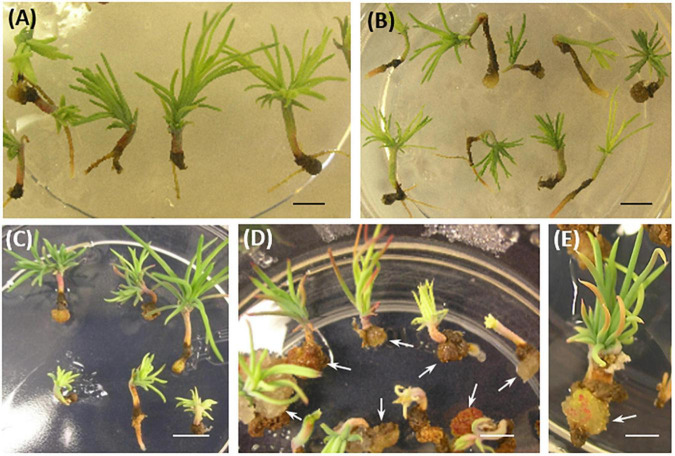
*P. pinaster* somatic seedlings from *35S::PpWOX2* transgenic lines and controls (EV-pC05, WT) after 8–10 weeks in conversion medium. **(A)** WT and **(B)** EV-pC05 somatic seedlings with similar and normal phenotype. The root growth of somatic seedlings from the *35S::PpWOX2* lines was reduced **(C,D)** and ultimately affected SE viability and behavior. Note the formation of callus in the root region of line #15 [**(D,E)**, arrows] compared to the WT and EV-pC05 controls **(A,B)**. Scale bars: **(A)** 2 mm, **(B–D)** 3 mm, and **(E)** 1 mm.

Somatic embryo (SE) viability was significantly reduced compared to controls after 8–10 weeks of culture in a conversion medium (Figure 5). Interestingly, line #15, which had the lowest ability for rooting, showed callus formation in the root region, and further root development was prevented in 73% of the somatic seedlings (Figures 3E, 5D,E).

To further examine the ability of transgenic plants overexpressing *PpWOX2* to initiate callus production, primary needle/hypocotyls and root explants from the *35S::PpWOX2*, EV-pC05, and WT somatic seedlings were cultured in an mLV medium without PGRs for 8 weeks. No direct somatic embryogenesis initiation could be detected under such experimental conditions. The calli obtained from the different types of explants from all lines were compact and white yellow, and did not differentiate into proliferating SEs (non-embryogenic calli).

**FIGURE 6:**
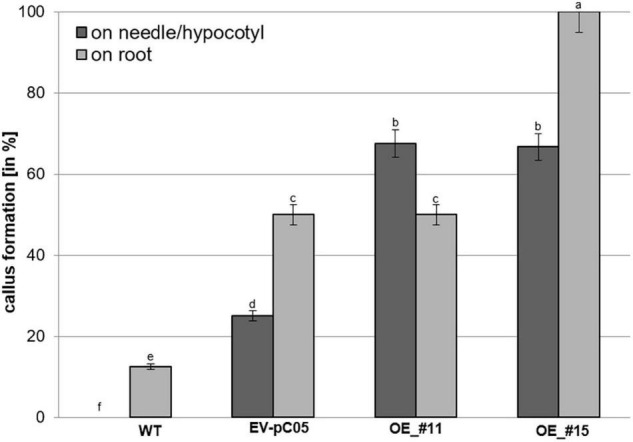
Percentage of non-embryogenic callus formation from *P. pinaster* primary needle/hypocotyl or root explants (*N* ≈ 20) of the *35S::PpWOX2* lines #11 and #15, transgenic EV-pC05, and non-transgenic WT controls in mLV medium without PGR (conversion medium). Each value represents mean ± standard error from three biological replicates and five Petri dishes as technical replicates. Bars with different letters indicate significant differences (ANOVA, *P* < 0.05).

The percentage of non-embryogenic callus formation from root explants was significantly higher in line #15 (100%) than in the WT (16%) and EV-pC05 (48%) controls [*F*(7,16) = 6.407, *P* = 0; Figure 6]. The observed increase in EV-pC05 compared to the WT explants was also significant and could indicate some habituation phenomenon of transgenic lines (following repeated subculture) to auxin supplemented in the proliferation medium. Nevertheless, this result shows that constitutive overexpression of *PpWOX2* in line #15 resulted in increased propensity to callus formation in the root pole, which, in turn, could be detrimental to root growth and subsequent plant viability (Figures 3E, 5). In the case of line #11, non-embryogenic callus formation (45%) was significantly increased compared to WT, but was not when compared to EV-pC05 [*F*(4,84) = 17.046, *P* = 0; Figure 6].

Nearly 65% of the needle/hypocotyl explants of both *35S::PpWOX2* lines #11 and #15 showed callus formation (Figure 6), and this was a significantly higher rate than that of the EV-pC05 (24%) and WT controls (0%). Similar to the root explants, EV-pC05 produced significantly more callus from needle/hypocotyl explants than the WT, suggesting, as mentioned earlier, some impact of the genetic transformation procedure.

Overall, the data showed that somatic seedlings of the *35S::PpWOX2* lines have an increased ability to initiate non-embryogenic callus on needle/hypocotyl only (line #11) or both needle/hypocotyl and root explants (line #15) compared to the non-transgenic WT and transgenic EV-pC05 controls.

### Phenotypic Response in Arabidopsis Plants Overexpressing PpWOX2

The overexpression of *PpWOX2* in *Arabidopsis* had different effects on the phenotype of transgenic (PCR+) seedlings from the T_1_ and T_2_ generations.

**FIGURE 7:**
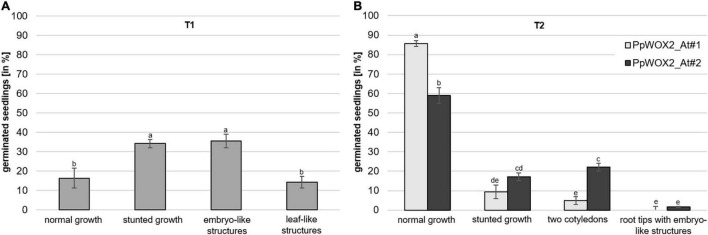
Frequency of different phenotypes among *PpWOX2* overexpressing *Arabidopsis* plants from the T_1_ or T_2_ generation. **(A)** T_0_ seeds transformed with the *35S::PpWOX2* gene cassette were germinated and resulting T_1_ plants (*N* = 70–80) developed different phenotypes (normal or stunted growth, embryo-like and leaf-like structures) in various frequencies. **(B)** T_1_ seeds transformed with the *35S::PpWOX2* gene cassette (*N* = 70–80) were germinated and resulting T_2_ plants from 2 independent transgenic lines (*PpWOX2*_At#1 and *PpWOX2*_At#2) developed either some phenotypes similar to those of T_1_ plants (normal growth and stunted growth) or new phenotypes not previously detected in the T_1_ plants (only two cotyledons and plants with root tips that formed embryo-like structures). Error bars indicate standard error for means of three biological replicates and 7–8 technical replicates. Bars with different letters show significant statistical differences (*P* < 0.05).

**FIGURE 8:**
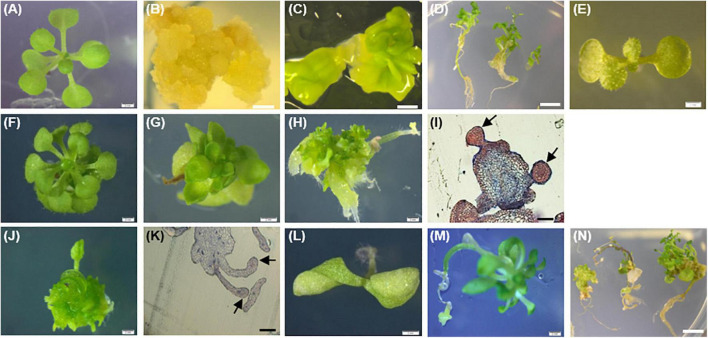
Phenotypes of the *35S::PpWOX2* transgenic plants, transgenic EV-pC05, and non-transgenic WT control plants of *Arabidopsis*. **(A)** WT seedling from first (T_1_) or second (T_2_) generation grown in MS medium without PGRs for 7 weeks. **(B)** WT non-embryogenic callus. **(C)** WT SEs obtained from explants of immature zygotic embryos cultivated in an induction medium (B5 medium supplemented with 5 μM 2,4-D) after 4 weeks. **(D)** WT SEs germinated and developed into somatic plants in MSR medium after 2 weeks. **(E)** EV-pC05 transgenic plant (transformed with the *pCAMBIA1305.2* empty vector) grown in MS medium supplemented with 20 mg l**^–^**^1^ hygromycin. These control plants exhibited a normal phenotype similar to that of WT seedlings **(A)** in both T_1_ and T_2_ generations. *PpWOX2*-transformed seeds of the T_1_ generation germinated in MS medium supplemented with 20 mg l**^–^**^1^ hygromycin and exhibiting **(F)** normal growth, **(G)** stunted growth, **(H)** embryo-like structures, or **(J)** leaf-like structures. Histological aspects of **(I)** embryo-like structures and **(K)** leaf-like structures (arrows) were analyzed after staining with Ponceau-methylene blue. The T_2_ generation of *PpWOX2* transgenic seeds developed into plants with **(F)** normal growth, **(G)** stunted growth, **(L)** two cotyledons, or **(M)** root tips forming embryo-like structures. **(N)** Embryo-like structure from the *PpWOX2* T_1_ plant generation converted into somatic plants in MSR medium. Scale bars: **(B,C,H,J)**, and **(L)** 0.5 mm; **(A,E,F,G)** 1 mm; **(D,N)** 1.2 mm; and **(I,K)** 0.2 mm.

Considering *PpWOX2*-expressing plants from the T_1_ generation, significant variation in the frequency of four classes of phenotypes were detected [*F*(3,8) = 9.74, *P* = 0.005, Figure 7A]. Only 16% of the *PpWOX2*-T_1_ transgenic seedlings showed the WT normal phenotype (Figures 8A,F), and the remaining 84% of the plants showed alterations such as stunted growth (34%, Figure 8G), embryo-like structures (35%, Figure 8H), and leaf-like structures (14%, Figure 8J). Histological examinations of the embryo-like structures (Figure 8H) revealed the presence of globular SEs with actively dividing cells (Figure 8I). Histochemical observation of the leaf-like structures (Figure 8J) only showed leaf anatomical characteristics without any evidence for embryo formation (Figure 8K).

In contrast to the T_1_ generation, a high percentage of the *PpWOX2*-expressing T_2_ plants showed normal (WT) growth with significant differences between the two investigated lines, *PpWOX2*_At#1 (85%) and *PpWOX2*_At#2 [60%, *F*(7,24) = 21.635, *P* = 0; Figure 7B]. Stunted growth was observed in only 9–15% of the plants without significant difference between the lines (Figure 7B). In addition, two new phenotypes were observed that did not appear previously in the T_1_ generation. First, a number of T_2_ seedlings (5–22%) were arrested in the two-cotyledon stage (Figure 7B), and no further leaf formation was observed (Figure 8L). This phenotype was found more frequently in *PpWOX2*_At#2 (22%) than in *PpWOX2*_At#1 (5%) lines [*F*(7,24) = 21.63, *P* = 0, Figure 7B]. Second, embryo-like structures suggesting direct somatic embryogenesis was observed at root tips of some plants from both *PpWOX2* transgenic lines (Figure 8M) but in quite low frequency (≤1.7%, Figure 7B).

The morphology and regeneration ability of embryo-like structures obtained from *PpWOX2* T_1_ plants (Figure 8H) were compared with that of SEs (Figure 8C) and non-embryogenic calluses (Figure 8B) initiated from the WT seedlings. Embryo-like structures and SEs were similar in their germination and regeneration abilities and phenotypes, as illustrated in Figure 8D (WT SEs) and 8N (*PpWOX2* plants derived from embryo-like structures), respectively. No plant regeneration could be obtained from non-embryogenic calluses.

The *PpWOX2* transgenic plants initiated flower formation earlier (12–15 days after germination) than the WT and EV-pC05 control plants (22–28 days). Control transgenic line EV-pC05 exhibited normal growth (a phenotype similar to that of WT-plants) in both the T_1_ and T_2_ generations (Figure 8E).

### Expression of Endogenous Embryogenesis-Related Genes in Arabidopsis Plants Overexpressing PpWOX2

**FIGURE 9:**
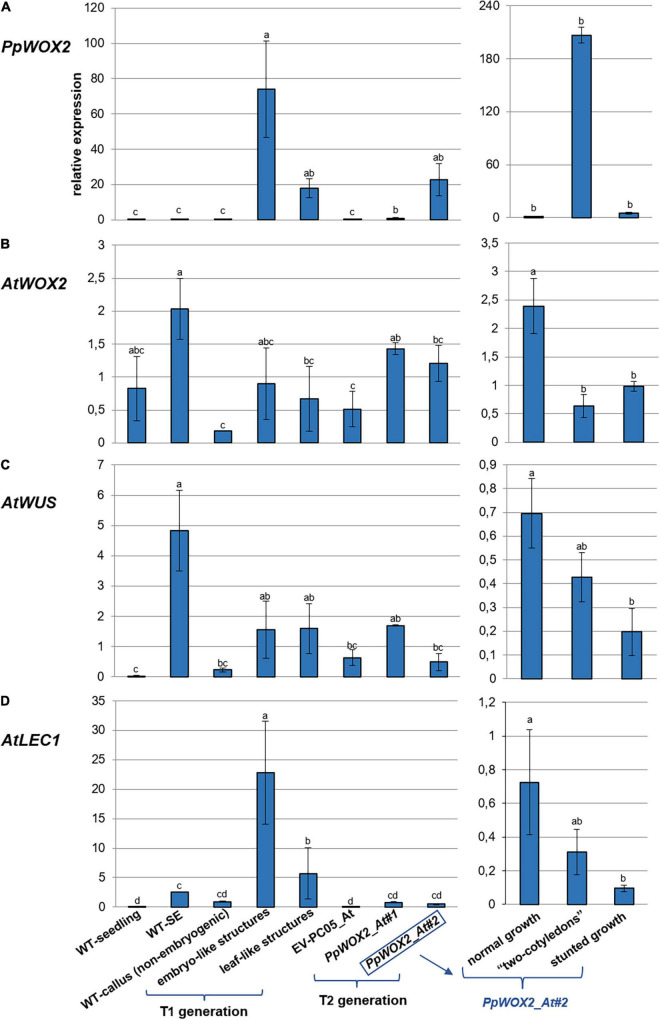
Relative expression of **(A)** *PpWOX2*, **(B)** *AtWOX2*, **(C)** *AtWUS*, and **(D)** *AtLEC1* in *Arabidopsis* plants obtained from *PpWOX2* overexpressing lines (T_1_ and T_2_ generations), transgenic EV-pC05_At, and non-transgenic WT controls (seedlings and somatic embryos/SE) at the same age and developmental stage by RT-qPCR. Each value represents mean ± standard error of three biological replicates and three technical replicates. Bars with different letters indicate significant differences (ANOVA, *P* < 0.05). The different phenotypes are illustrated in Figure 8. Samples *PpWOX2_*At#1 and *PpWOX2*_At#2 from the T_2_ generation are a mixture of plants with the “normal,” “two-cotyledon,” and “stunted growth” phenotypes. Gene expression is presented separately for these three phenotypes for line *PpWOX2_At#2* (right part of the figure).

In order to investigate whether the constitutive overexpression of *PpWOX2* in *Arabidopsis* affects the expression of selected embryogenesis-related endogenous genes, the expression of *AtWOX2*, *AtWUS*, and *AtLEC1* was estimated by RT-qPCR in *35S::PpWOX2* T_1_ and T_2_ transgenic lines exhibiting different phenotypes (Figure 9). Controls were the empty vector transgenic line (EV-pC05_At) and various non-transgenic lines with unknown (WT seedlings), demonstrated (WT SE), or no (non-embryogenic callus) embryogenic ability. All analyzed tissues (excluding non-embryogenic callus) were sampled from plants of the same age and developmental stage.

The relative expression of *PpWOX2* was confirmed to be significantly higher in transgenic *Arabidopsis* plants of both T_1_ and T_2_ generations than in transgenic (EV-pC05_At) and non-transgenic (WT) controls [*F*(7,16) = 3.882, *P* = 0.012; Figure 9A]. Among the different phenotypes observed for the T_1_ and T_2_ plants, expression was similar and lowest for plants showing normal and stunted growth as illustrated for the *PpWOX2*_At#2 line (Figure 9A, right graph). *PpWOX2* expression was much higher in T_1_ plants showing embryo- or leaf-like structures than in transgenic plants with normal growth (10- to 35-fold ratio, data not shown). Similarly, *PpWOX2* expression was significantly higher in T_2_ plants with the “two-cotyledon” phenotype for both lines *PpWOX2*_At#1 (data not shown) and *PpWOX2*_At#2 (128-fold ratio, Figure 9A, right graph). The reduced behavior of T_2_ transgenic plants with root tips showing an embryo-like structure did not allow for gene expression analysis.

In contrast to *PpWOX2*, the expression of *AtWOX2* (Figure 9B) was mostly similar in the T_1_ plants and controls (Figure 9B). Only plants with the “leaf-like structure” phenotype showed significantly reduced *AtWOX2* expression compared to the non-transgenic SE control. Considering the T_2_ plants, some significant differences were observed for the *PpWOX2*_At#1 line, which showed higher *AtWOX2* expression than the EV-pC05_At and non-embryogenic callus controls. However, the expression was similar to that of control non-transgenic seedlings and SE (WT). Similarly, no significant difference compared to controls could be detected in the case of line *PpWOX2*_At#2 when all plant phenotypes were considered together as a mixture. Analyzing separately the different plant phenotypes for this line revealed that *AtWOX2* expression is similarly and significantly reduced in plants with two cotyledons or stunted growth compared to plants with normal growth (Figure 9B, right graph). We concluded that *PpWOX2* overexpression had apparently little effect on the expression of endogenous *WOX2*. The *PpWOX2*_At#2 data for plants with normal, stunted, or two-cotyledon phenotypes, however, suggest some inverse relationship between *PpWOX2* (Figure 9A) and *AtWOX2* expressions (Figure 9B).

In the case of *AtWUS* expression (Figure 9C), some significant differences with non-transgenic control seedlings were detected for T_1_ plants and T_2_ plants from the *PpWOX2*_At#1 line (higher expression than in WT seedlings). *AtWUS* expression in T_2_ plants from the *PpWOX2*_At#2 line was also significantly lower than in non-transgenic control SEs. However, *AtWUS* expression was found to be similar in both T_1_ and T_2_ *PpWOX2* transgenic plants and EV-pC05_At transgenic control, suggesting that *PpWOX2* overexpression had no drastic effect on the expression of this gene. *AtWUS* expression was found to be higher in T_2_ plants from line *PpWOX2*_At#2 exhibiting normal growth (significant) and “2 cotyledon” phenotype (non-significant) compared to plants with “stunted” phenotype (Figure 9C, right graph). However, there is no clear correlation with *PpWOX2* transgene expression.

Expression of *AtLEC1* is significantly higher in T_1_ plants with embryo-like and leaf-like structures than in all the controls (Figure 9D). In contrast, no significant difference could be detected in the case of T_2_ plants. However, considering line *PpWOX2*_At#2, *AtLEC1* expression (Figure 9D, right graph) showed a pattern similar to that for *AtWUS* (Figure 9C, right graph), i.e., higher expression in plants with normal growth (significant) and “two-cotyledon” phenotype (non-significant) compared to plants with stunted growth.

In brief, high *PpWOX2* expression in T_1_ transgenic plants with embryo-like or leaf-like structures is correlated with increased expression of *AtLEC1* but not *AtWOX2* or *AtWUS*. High *PpWOX2* expression in T_2_ transgenic plants with the “2-cotyledon” phenotype is correlated with decreased expression of *AtWOX2*.

## Discussion

In angiosperms, the transcription factor *WOX2* is necessary for cell fate and delineation of the apical embryo domain during embryogenesis (Haecker et al., 2004; Xiao et al., 2006). Zhang et al. (2017) demonstrated that *WOX2* contributes to the initiation of shoot meristem stem cells in the embryo of *Arabidopsis*. Several reverse genetic studies have been reported on knockout and overexpression of *WOX2* in angiosperms, mainly in *Arabidopsis* (Haecker et al., 2004; Jeong et al., 2011; Elhiti et al., 2013; Chung et al., 2016; Zhang et al., 2017). For example, loss of *AtWOX2* function in zygotic embryos of *Arabidopsis* has only relatively mild consequences and results in aberrant divisions at the apex of the globular pro-embryo and, occasionally, seedlings with a single cotyledon (Haecker et al., 2004; Jeong et al., 2011). In contrast, overexpression of *AtWOX2* in *Arabidopsis* causes severe growth defects and further morphological phenotypes by impairing plant organ formation (Chung et al., 2016).

In conifers, much attention has been paid to identify genes that are important during embryogenesis [Park et al., 2010; Rupps et al., 2016; reviewed in Cairney and Pullman (2007), Miguel et al. (2016), Trontin et al. (2016a)] but a considerable number of studies are still needed to unravel the precise spatiotemporal function of each gene. Zhu et al. (2016) reported that downregulation of *WOX2* of *Picea abies* (*PaWOX2*) during early embryogenesis resulted in significant decrease in the yield of mature embryos. In contrast, downregulation of *PaWOX2* after late embryo formation had no effect on further embryo development and maturation. In this study, we focused on the functional study on a *WOX2* gene isolated from *P. pinaster* (*PpWOX2*) by constitutive overexpression of *PpWOX2* in both *P. pinaster* SEs and *Arabidopsis* seedlings.

The deduced WOX2 protein shares the WUS-type homeodomain and the conserved WUS-box of the WOX family proteins (Figure 1). Based on phylogenetic analyses, the 15 members of the WOX family are divided into three clades: the ancient clade, the intermediate clade, and the WUS clade (modern clade) (van der Graaff et al., 2009; Hedman et al., 2013; Lian et al., 2014; Alvarez et al., 2018). The modern clade contains *WUS* and *WOX1-7* members that are found in seed plants, confirming an evolutionary relationship among *WOX* genes of this group (Lian et al., 2014; Alvarez et al., 2018). *PpWOX2* shares high identity to its homolog in *Pinus sylvestris* (97%) and *Pinus contorta* (95%), low global identity to the *WOX2* of *Picea abies* (76%) and *Larix decidua* (63%), and very low identity to the *WOX2* of *Arabidopsis* (35%) (Hedman et al., 2013; Zhu et al., 2016).

The expression of endogenous *PpWOX2* in *P. pinaster* is high in the SE proliferation step but significantly decreases after SE maturation. No *PpWOX2* expression could be detected later in somatic seedlings. These data confirmed (SE development) and extend (somatic plant development) previous results obtained by Alvarez et al. (2018) from a different *P. pinaster* embryogenic line and following a different protocol for somatic embryogenesis. A similar *WOX2* expression pattern has been found in other conifers such as *Larix decidua* (Rupps et al., 2016), *Picea abies* (Palovaara et al., 2010a; Hedman et al., 2013), and *Picea glauca* (Klimaszewska et al., 2011). These differences in *WOX2* expression during SE development may be explained by an internal control of *PpWOX2* by other genes that are differentially expressed during development, such as polar auxin transport-related genes (PIN genes) (Palovaara and Hakman, 2009). Considering these expression data, it would be interesting to confirm that *PpWOX2* can be used in *P. pinaster* to distinguish in the proliferation stage embryogenic from non-embryogenic tissues. In conifers, the expression of *WOX2* is indeed known as a putative marker for effective initiation of somatic embryogenesis and to predict the embryogenic potential (embryogenecity) of a culture (Palovaara and Hakman, 2008; Park et al., 2010; Klimaszewska et al., 2011; Miguel et al., 2016).

Over-expression of *PpWOX2* in proliferating SEs of *P. pinaster* had, apparently, a negative effect on maturation (Figure 3B). Both lines, OE_#11 and OE_#15, were found to have a significant lower maturation ability than the non-transgenic WT control. These results are consistent with those reported by Klimaszewska et al. (2010). In this report, it was shown that induced overexpression of *AtWUS* in SEs of *Picea glauca* had a striking effect on SE maturation by disrupting embryo development. According to this result, it may be postulated that *WOX* genes of (at least) the modern clade influence embryo development in conifers. However, embryos of the empty vector control line (EV-pC05) also showed significant decrease in maturation yield in our experiments compared to non-transgenic WT embryos, suggesting that reduced yield could also result from transgene positional effects and/or technical issues such as differences in physiological aging between transgenic lines and WT controls (discussed in Supplementary Table 1). In maritime pine, physiological aging of embryogenic lines results in reduced maturation yield (Breton et al., 2006; Lelu-Walter et al., 2016; Trontin et al., 2016b). Aging effects could, therefore, result from unperceived differences in management of transgenic and WTs during the long process of genetic transformation. The development of a simplified, rapid, and improved genetic transformation protocol of embryogenic tissues would be profitable to reduce possible protocol-related effects on maturation yield. Furthermore, the choice of a selective agent may influence maturation results. Hygromycin is known to affect negatively maturation yield in *P. pinaster* when used at 20 mg l^–1^ (Trontin et al., 2007). Although hygromycin concentration was reduced to 10–15 mg l^–1^ in our experiments, hygromycin could have affected the regeneration of transgenic embryos of the *PpWOX2* and control EV-pC05 lines.

The overexpression of *PpWOX2* in *P. pinaster* negatively affected SE development with i) significantly increased frequency of deformed SE, especially asymmetrical cotyledon development (lines OE_#11 and OE_#15, Figure 3C) and ii) non-embryogenic callus formation in the root pole of germinating somatic seedlings, with subsequent inhibition of root growth (especially in the highly overexpressing line OE_#15, Figures 3E, 5D,E). Similarly, Klimaszewska et al. (2010) reported the inhibition of root growth in somatic seedlings of *Picea glauca* after induced overexpression of *AtWUS* during SE conversion. Following downregulation of *WOX2* during early embryogenesis in *Picea abies*, a unique function of *WOX2* was suggested in conifers during protoderm development and suspensor expansion, which are important steps for proper early embryo development (Zhu et al., 2016).

To further investigate the propensity of young *P. pinaster* somatic seedlings transformed with *PpWOX2* by callus formation, needle/hypocotyl and root explants from two transgenic lines were cultured in the mLV medium (Litvay et al., 1985) without PGRs. No formation of embryogenic tissue could be detected from both transgenic lines and controls, suggesting that *PpWOX2* constitutive overexpression had no major and direct effect that could stimulate embryogenic potential in young somatic seedlings without PGRs. mLV is the currently preferred basal medium for *P. pinaster* to initiate somatic embryogenesis from immature zygotic embryos (with or without PGRs, Trontin et al., 2016b) and secondary embryogenesis from cotyledonary SEs and somatic seedlings (with PGRs, Klimaszewska et al., 2009). Furthermore, mLV is useful to induce non-embryogenic callus formation from young to more mature materials with or without PGRs (Trontin et al., 2016b,c). Therefore, we initially considered that the overexpression of *PpWOX2* could be sufficient to stimulate the formation of embryogenic tissues from somatic seedling explants. However, as in most conifer species (see Bonga et al., 2010), initiation of somatic embryogenesis in *P. pinaster* materials older than immature zygotic embryos (Trontin et al., 2016c) or cotyledonary or young germinating SEs (Klimaszewska et al., 2009) is still difficult to achieve and may require the additional use of PGRs such as auxin and/or cytokinins. Recently, it was shown that abscisic acid (ABA) supplementation can also lead to initiation of somatic embryogenesis in the Douglas fir (*Pseudotsuga menziesii*) (Walther et al., 2021) and has to be taken into consideration as a signal for SE activation. Induction of secondary somatic embryogenesis from germinated SEs could be obtained but at low rate with a combination of 2,4-D (9.5 μM) and BA (4.5 μM) in *P. pinaster* (Klimaszewska et al., 2009). In *Capsicum annuum*, ectopic *BABY BOOM* expression is not sufficient to induce embryogenesis, and exogenous cytokinin is required for SE formation (Heidmann et al., 2011). However, ectopic expression of *BABY BOOM* in *Arabidopsis* and *Brassica* led to spontaneous formation of SEs and cotyledon-like structures in seedlings without the use of exogenous PGRs (Boutilier et al., 2002). The enhanced embryogenic potential of young somatic seedlings in *P. pinaster* may require a combination of ectopic expression of embryogenesis-related gene(s) and adequate PGR treatment and/or other environmental conditions.

Instead of embryogenic callus, non-embryogenic callus formation was determined with high frequency in needle/hypocotyl (significant in both lines #11 and #15) and/or root explants (significant in line #15) of *PpWOX2* somatic seedlings (Figure 6) cultivated in mLV deprived of PGRs. Non-embryogenic callus formation was similarly significantly enhanced in the transgenic control EV-pC05 line compared to the WT, suggesting some technical issues related with genetic transformation. However, this increase was lower than that observed for the *35S::PpWOX2* lines except in the case of line #11 for the root explants. These results may be explained by alteration in levels of endogenous hormones, particularly auxin, in response to *PpWOX2* overexpression in somatic seedlings. Recent studies showed that at the time of stem cell initiation, *WOX2* affects the auxin pathway by increase in expression of the auxin transporter *PIN1* gene (Zhang et al., 2017). In conifers, Palovaara and Hakman (2009) suggested that polar auxin transport is involved in regulation of the expression of both the auxin efflux carrier (encoded by *PIN1*) and *WOX2*. N-1-naphthylphthalamic acid (NPA) treatment of embryos before cotyledon initiation, indeed, disrupted the endogenous auxin pattern and expression of both *PIN1-like* and *WOX2* (Hakman et al., 2009). Interestingly, the expression of *PIN* homologs was associated with the auxin immunolocalization pattern during cotyledon formation in *Picea abies* (Palovaara et al., 2010b). It was suggested that correct auxin transport is crucial during transition from early to pre-cotyledonary embryos and that it is involved in the coordinated regulation of *WOX2* and *PIN1* (Trontin et al., 2016a), which, in turn, could affect both cotyledonary embryo development (especially cotyledon formation) and normal root formation. Further analysis is needed to consider the interplay of *PpWOX2* and auxin in *P. pinaster* and other conifers.

Furthermore, the heterologous overexpression of *PpWOX2* in *Arabidopsis* resulted in transgenic seedlings (T_1_ and T_2_ generations) with different phenotypes such as normal growth, stunted growth, and embryo-like and leaf-like structures. One-third (35%) of the first generation of *PpWOX2*-At plants showed embryo-like structures. In addition, a similarly high number of T_1_ plants exhibited stunted growth (35%) and leaf-like structures (15%). *PpWOX2* expression was comparatively low in T_1_ plants exhibiting a normal growth phenotype (similar data to “normal growth”-type of T_2_ plants, Figure 9A, right graph). In different phenotypes of T_2_ plants, reduced *PpWOX2* expression in the normal phenotype compared to stunted- and even more (significant) two-cotyledon phenotypes (Figure 9A) apparently resulted in increase in frequency of the normal phenotype in T_2_ plants compared to the other phenotypes (Figure 7B), while a positive link between the expression of *PpWOX2* and the frequency of abnormal phenotypes was observed in the T_2_ plants. Similarly, the overexpression of *PpWOX2* in immature SEs of *P. pinaster* (Figure 3A) is correlated to decrease in the frequency of normal, well-developed, cotyledonary embryos compared to the WT (Figure 3B), although not significant in our experiments when compared to the EV-PC05 transgenic control. Overall, these results suggest that the high, constitutive expression of *PpWOX2* negatively affects the frequency of the normal phenotype in both *Arabidopsis* and *P. pinaster*.

*PpWOX2* expression is higher in the transgenic T1 and T2 plants than in the WT control material (seedlings, SEs, non-embryogenic callus) and transgenic control EV-pC05 (Figure 9A and Supplementary Table 1). However, the different phenotypes observed in transgenic T1 and T2 plants (Figures 7, 8) are only weakly supported by gene expression data (e.g., lower *PpWOX2* expression in the T2 plants with normal and stunted growth than in the “two-cotyledon” plants, Figure 9). Some post-transformation silencing effects related to 35S promoter inactivation (a well-known effect during transgenesis) may be partially responsible for such discrepancy. It can be suspected that transgene inactivation (and associated phenotype reversion) could be high in the T2 generation as the frequency of plants with normal phenotype is significantly higher (60–85%) than in T1 (15–20% only). Different *PpWOX2* expression levels revealed by RT-qPCR may, therefore, not reflect the actual levels of PpWOX2 protein production. Our data do not allow for a study on whether transcriptional or post-transcriptional silencing mechanisms could be involved. However, considering the high levels of *PpWOX2* expression observed in some plants with abnormal phenotypes, such as the two-cotyledon T2 plants (At#2), and embryo-like and leaf-like T1 structures (Figure 9A), it could largely be an effect of post-transcriptional or even translational regulation of *PpWOX2* expression. There is generally low support in our experiments for an effect of *PpWOX2* overexpression (Figure 9A) on the germination rate of T1 and T2 plants (Figure 7). Observed data may be more related with various degrees of silencing, as only T2 plants with normal phenotype germinated with excellent rates (similar to those of WT seedlings).

Moreover, in *Arabidopsis*, the embryo-like and leaf-like phenotypes in T_1_ plants, persistence of the stunted growth phenotype in T_1_ (35%) and T_2_ plants (up to 15%), and production of T_2_ plants with two cotyledons (5–20%) or formation of embryo-like structures on the root tip (1.7%) directed us to analyze the endogenous expression of several major embryogenesis-related genes (*AtWOX2*, *AtLEC1*, and *AtWUS*) to investigate a putative association with observed phenotypes. As hundreds of genes are probably involved in *Arabidopsis* embryogenesis, an extension of this expression study and additional transcriptomic and/or proteomic approaches are required to gain further insight into intrinsic processes (Tzafrir et al., 2004; De Smet et al., 2010; Elhiti et al., 2013).

*PpWOX2* constitutive over-expression in *Arabidopsis* had a globally low effect on endogenous *AtWOX2* and *AtWUS* expressions, which are roughly similar in transgenic lines and controls from the T_1_ and T_2_ generations.

The expression of *AtLEC1* was much more affected, especially in the T_1_ generation (significant overexpression). Indeed, transgenic *PpWOX2* plants from the T_1_ generation with embryo-like and leaf-like structures showed very high expression of *AtLEC1* compared to the WT non-transgenic (seedlings, SEs, and non-embryogenic callus) and transgenic (EV-pC05_At) controls. These strong results suggest that overexpression of *PpWOX2* in *Arabidopsis* may stimulate embryogenesis and organogenesis through coordinated overexpression of *AtLEC1*. Consistent with this outcome, previous reports support that the over-expression of *AtLEC1* in *Arabidopsis* induced somatic embryogenesis in vegetative cells (Lotan et al., 1998).

In addition, the expression of *PpWOX2* in transgenic plants with embryo-like and leaf-like structures was much higher (10 − 35-fold) than in transgenic plants with normal growth. Therefore, a plausible explanation is that ectopic expression of *PpWOX2* in *Arabidopsis* might act redundant with *AtWOX*2 in inducing embryogenesis and/or organogenesis. Another possible explanation is that the overexpression of *PpWOX2* in *Arabidopsis* might result in the over-expression of *AtWUS*, which activates the expression of *LEC1* and promotes somatic embryogenesis. It was reported that *AtWUS* promoted somatic embryogenesis and activated the expression of *LEC1*, *LEC2*, and *FUS3* in cotton (Zheng et al., 2014). The authors suggested that *AtWUS* may alter *PIN* expression, which could lead to establishment of new auxin gradients. Subsequently, a new auxin response was formed and stimulated the expression of *LEC1*, *LEC2*, and *FUS3*. In addition, the overexpression of *GgWUS* from *Gnetum gnemon* (Coniferopsida) in *Arabidopsis* was observed to induce somatic embryogenesis and organogenesis (Nardmann et al., 2009). In fact, *WUS* is involved in regulation of both meristematic stem cells (pluripotent) and embryogenic stem cells (totipotent) (Elhiti et al., 2013). Moreover, *WUS* is known as an embryogenesis marker in embryonic cells, and its overexpression in *Arabidopsis* resulted in somatic embryogenesis and repeated formation of adventitious shoots in the absence of auxin (Zuo et al., 2002; Elhiti, 2010). We did not obtain clear results of *AtWUS* expression during our experiments. The data showed some high expression of *AtWUS* compared to the non-transgenic WT seedling control, but the differences were not significant compared with the other non-transgenic (WT-SE, WT-callus) and transgenic (EV-pC05_At) controls. The heterologous expression of *PpWOX2* in *Arabidopsis* (which only has 35% similarity with *AtWOX2*, Figure 1) cannot apparently direct increased expression of either *AtWUS* (Figure 9C) or *AtWOX2* (Figure 9B), which, in turn, could affect the expression of *AtWUS* and/or *AtLEC1*. Specific experiments are, therefore, needed to confirm this hypothesis.

Considering the emergence of various phenotypes in the T_2_ generation following *PpWOX2* overexpression, our data showed that the normal/abnormal plant phenotype is associated with altered *AtWOX2*, *AtWUS*, and *AtLEC1* expression (*PpWOX2_At#2*). It is suggested that variable levels of ectopic overexpression of *PpWOX2* could affect the expression of these three embryogenesis-related genes.

One possible further explanation on how overexpression of *PpWOX2* in *Arabidopsis* can stimulate somatic embryogenesis and organogenesis is the high similarity of the *WUS* family-specific homeodomain and the WUS-box from PpWOX2 and AtWOX2 proteins. In plants, the *WOX* homeobox is assigned to a subfamily of homeobox transcription factors that are involved in plant embryonic patterning. However, the function of the *WOX* homeobox has not yet been analyzed in plants. In animals, it is confirmed that a homeobox gene family (HOX), which is expressed in specific embryo domains, has a major regulatory role during early pattern formation, similar to that of the WOX homeobox family (Haecker et al., 2004). In this study, the complete protein sequences of PpWOX2, AtWOX2, and AtWUS show low similarity (35%), but they share high similarity (78%) in the WUS-type homeodomain and WUS-box. Wu et al. (2007) suggested that functional redundancy in the WOX family was not solely determined by overall protein sequence similarity but by high similarities of the homeodomains.

Thus, it can be concluded that high similarities of the WUS-type homeodomain and WUS-box among *PpWOX2*, *AtWOX2*, and *AtWUS* might be sufficient for functional redundancy in these *WOX* proteins. This speculation is supported by several lines of evidence:

First, the homology of *WOX* homeodomains among the *WOX* genes supports three major clades: the ancient, intermediate, and modern clades. According to phylogenetic analysis, the *WOX1-7* and *WUS* genes are grouped in the modern clade. Some subgroups from the modern clade have been lost in several species (e.g., lack of WOX1/6 subgroup in rice). These studies imply that *WOX* members in the modern clade may have a conserved and redundant function (Hedman et al., 2013; Lian et al., 2014; Alvarez et al., 2018).

Second, in angiosperms, the expression of *WUS* and *WOX5* is specific to the shoot and root regions, respectively. Furthermore, Sarkar et al. (2007) demonstrated that *WOX5* and *WUS* are interchangeable for stem cell control in *Arabidopsis*. In contrast, Nardmann et al. (2009) reported that *Ginkgo biloba WUS* (*GbWUS*) and *Pinus sylvestris WUS* (*PsWUS*) are expressed in both the shoots and the roots, and suggested that the *WUS* and *WOX5* genes are the result of an angiosperm-specific gene duplication.

## Conclusion

Our results suggest that constitutive overexpression of *PpWOX2* in *Arabidopsis* seedlings alters the expression of embryogenesis-related genes (*AtLEC1* and/or *AtWUS*), which, in turn, could promote the formation of SEs and organs. Further reverse genetics studies through inducible overexpression of combinations of embryogenesis-related genes and possibly an additional (hormonal) stimulus might be helpful to overcome recalcitrance to somatic embryogenesis of already differentiated tissues in *Pinus pinaster*. These findings might be helpful to gain insights into conifer embryogenesis and, in best case, to develop strategies for induction of somatic embryogenesis from adult conifer trees (Lelu-Walter et al., 2016; Trontin et al., 2016c).

## Data Availability Statement

The datasets presented in this study can be found in online repositories. The names of the repository/repositories and accession number(s) can be found below: https://www.ncbi.nlm.nih.gov/, KY773924.1.

## Author Contributions

AR, KZ, JR, and SH conceived and designed the research project with expert guidance from J-FT for the maritime pine work. J-FT provided PN519 as starting material for maritime pine. J-FT and AR provided the plasmids and methods for the plant transformation experiments. SH and AR conducted the experiments, analysed the data and wrote the manuscript. KZ and AR supervised the lab work. All authors reviewed and approved the final version of the manuscript.

## Conflict of Interest

The authors declare that the research was conducted in the absence of any commercial or financial relationships that could be construed as a potential conflict of interest.

## Publisher’s Note

All claims expressed in this article are solely those of the authors and do not necessarily represent those of their affiliated organizations, or those of the publisher, the editors and the reviewers. Any product that may be evaluated in this article, or claim that may be made by its manufacturer, is not guaranteed or endorsed by the publisher.

## Footnotes

1
https://www.scbi.uma.es/sustainpinedb

2
http://www.procogen.eu

3
http://www.nasc.co.uk

4
www.cambia.org
