# Supplementary material for: Constitutive Overexpression of a Conifer WOX2 Homolog Affects Somatic Embryo Development in Pinus pinaster and Promotes Somatic Embryogenesis and Organogenesis in Arabidopsis Seedlings
Source: Front Plant Sci. 2022 Mar 10;13:838421. doi: 10.3389/fpls.2022.838421 (PMC8960953; doi:10.3389/fpls.2022.838421)
Supplement: Supplementary file 1 [file Table_1.pdf]

Supplementary Table 1. A summary of observed effects following constitutive overexpression of *PpWOX2* in maritime pine embryogenic lines PN519 or Arabidopsis ecotype Columbia-0, and tentative interpretation of the origin of such effects (technical issue and/or transgene effect).

**Target species for *PpWOX2* constitutive overexpression**	**Observed effect in transgenics compared to WT material**	**Observed effect in transgenic EV-pC05 control compared to WT material**	**Origin of observed effects (tentative)**
P. pinaster (PN519 embryogenic line)	Higher *PpWOX2* expression in proliferating SE, mature SE and somatic seedlings (**Figure 3A**)	No comparison provided	Unable to distinguish between putative technical and transgene effects. Similar results obtained for 10 cryopreserved *35S::PpWOX2* lines.
	Reduced yield in mature, cotyledonary SE (**Figure 3B**)	Reduced yield (**Figure 3B**)	Rather a technical outcome: physiological ageing of transgenic lines and/or adverse effect of the hygromycin selective antibiotic (Trontin et al. 2007).
	Increased frequency of deformed mature embryos (**Figure 3C**)	No significant difference (**Figure 3C**)	*PpWOX2* overexpression effect detected
	Similar conversion rates of mature embryos into somatic seedlings (**Figure 3D**)	Similar conversion rates of mature embryos into somatic seedlings (**Figure 3D**)	No technical outcome or transgene effect detected
	Trend towards reduced root growth in somatic seedlings, only significant for line OE_#15 (**Figure 3E**)	Slightly reduced root growth in somatic seedlings but no significant difference (**Figure 3E**).	*PpWOX2* overexpression effect detected. Technical outcome cannot be excluded but is not detected in our experiments.
	Reduced somatic seedling viability after 8-10 weeks culture on conversion medium	No significant difference	Rather an effect of *PpWOX2* overexpression in relation to reduced root growth in transgenics (**Figure 3E**).
	Increased propensity to produce non-embryogenic callus from needle/hypocotyl and root seedling explants (**Figure 6**)	Increased propensity to produce non-embryogenic callus from needle/hypocotyl and root seedling explants (**Figure 6**)	Large technical outcome detected. As the ability of transgenic lines to produce non-embryogenic callus is also significantly increased compared to EV-pC05 transgenic control (except line OE_#11 for root explants), a *PpWOX2* overexpression effect is also detected and superimposed.
A. thaliana (ecotype Columbia-0)	Set of abnormal phenotypes in T1 and T2 plants (**Figures 7, 8**)	No significant difference	*PpWOX2* overexpression effect detected
	Earlier flowering of T1 and T2 plants (12-15 days after germination)	No significant difference	*PpWOX2* overexpression effect detected

Higher *PpWOX2* expression in abnormal T1 (embryo-like structures, leaf-like structures) and T2 plant mixtures (normal, two-cotyledons and stunted growth phenotypes) (**Figure 9A**)	No significant difference (**Figure 9A**)	*PpWOX2* overexpression effect detected
Similar *AtWOX2* expression pattern of T1 and T2 plants compared to WT-seedlings and non-embryogenic WT callus (**Figure 9B**)	No significant difference with WT-seedlings and non-embryogenic WT callus (**Figure 9B**)	No technical outcome or transgene effect detected
Lower *AtWOX2* expression in abnormal T1 leaf-like structures compared to WT-SE (**Figure 9B**)	Lower *AtWOX2* expression compared to WT-SE (**Figure 9B**)	Putative technical outcome
Higher *AtWUS* expression in T1 and T2 plants compared to WT-seedlings (not significant for PpWOX2_At#2 plant mixture, **Figure 9C**).	No significant difference compared to WT-seedling (**Figure 9C**)	Putative *PpWOX2* overexpression effect
Similar *AtWUS* expression pattern of T1 and T2 plants compared to WT-SE and non-embryogenic WT callus (**Figure 9C**) except for plant mixture PpWOX2_At#2 (reduced compared to WT-SE)	Lower *AtWUS* expression compared to WT-SE but similar compared to non-embryogenic WT callus (**Figure 9C**)	Either no *PpWOX2* effect or putative technical outcome
Higher *AtLEC1* expression in T1 plants (all WT controls, **Figure 9D**)	No significant difference (compared to WT-seedling and non-embryogenic WT-callus) or reduced expression of *AtLEC1* compared to WT-SE (**Figure 9D**)	*PpWOX2* overexpression effect detected
Similar *AtLEC1* expression in T2 plants (all WT controls, **Figure 9D**)	No significant difference (compared to WT-seedling and non-embryogenic WT-callus) or reduced expression of *AtLEC1* compared to WT-SE (**Figure 9D**)	No transgene effect detected

Notes: *AtWOX2*, *AtWUS*, *AtLEC1*: *Arabidopsis thaliana WOX2*, *WUS* and *LEC1* genes, respectively; EV-pC05: control line transformed with pCAMBIA1305.2 empty vector; *PpWOX2*: *Pinus pinaster WOX2* gene; SE: somatic embryos; T1/T2: first- and second-generation plants, respectively; WT: wild type;

Cited reference

Trontin, J.-F., Walter, C., Klimaszewska, K., Park, Y. S., and Lelu-Walter, M. A. (2007). Recent progress in genetic transformation of four *Pinus* spp. Transgenic Plant J. 1, 314–329.
